# Investigation of a family affected by early-onset osteoarthritis – proposal of a clinical pathway and bioinformatics pipeline for the investigation of cases of familial OA

**DOI:** 10.1186/s12891-023-06691-5

**Published:** 2023-07-13

**Authors:** Leticia A. Deveza, Andreas Zankl, David J. Hunter

**Affiliations:** 1 Sydney Musculoskeletal Health, Kolling Institute, Faculty of Medicine, University of Sydney, Camperdown, Australia; 2 Rheumatology Department, Royal North Shore Hospital, Reserve Road, St Leonards, NSW 2065 Australia; 3 Department of Clinical Genetics, The Children’s Hospital at Westmead, Hawkesbury Road, Westmead, NSW Australia; 4 Sydney Medical School, The University of Sydney, Camperdown, NSW Australia; 5 Bone Biology Division and Kinghorn Centre for Clinical Genomics, Garvan Institute of Medical Research, Darlinghurst, NSW Australia

**Keywords:** Osteoarthritis, Dysplasia, Arthritis

## Abstract

**Background:**

Familial cases of early-onset osteoarthritis (OA) are rare although the exact prevalence is unknown. Early recognition of underlying OA-associated disorders is vital for targeted treatment, when available, and genetic counselling, in case of skeletal dysplasias. Currently, there is no clear guidance on how best to investigate families affected by early-onset OA.

**Methods:**

We investigated a family with multiple members affected by early-onset OA (age at onset ≤ 40 years). Clinical and demographic characteristics were collected, followed by laboratory investigations screening for a range of potential OA-associated disorders, and whole genome sequencing in selected individuals.

**Results:**

Seventeen members of the family were included (7 affected and 10 non-affected). There was an even split between the two sexes and two participants were under 18 years old. No pattern of abnormality was seen in the laboratory investigation that could explain the OA phenotype in the family. Whole-genome sequencing was perfomed in one participant and analysed for likely pathogenic variants in genes known to be associated with skeletal dysplasias. A heterozygous variant in the *COL2A1* gene was identified (p.Arg519Cys). Confirmatory tests were performed in five additional participants (four affected and one unaffected).

**Conclusion:**

The methodology used in this study, including the clinical pathway and bioinformatics pipeline, could be applied to other families affected by early-onset OA.

## Introduction

Osteoarthritis (OA) is an incredibly prevalent joint disease with a higher prevalence among older adults and overweight/obese individuals [1], reaching 23 to 32% in obese men and women, respectively, above 85 years [2]. Although the pathogenic mechanisms of OA have not been fully elucidated, the condition appears to be a result of an active and complex process involving inflammatory, mechanical and metabolic factors that ultimately lead to the structural destruction of the joint [3]. Quality of life and activity participation are often compromised. Around 40% of those diagnosed with arthritis, among which OA is the most common cause, are estimated to have arthritis-attributable activity limitations [4]. Current OA treatment is largely aimed at alleviating symptoms since there is no treatment that has been proven to halt the disease progression or restore joint health.

Osteoarthritis is a multifaceted syndrome with a wide range of risk factors, including older age, obesity, joint injury, abnormal joint shape and alignment, hormonal changes and genetic predisposition. Occasionally, specific diseases can cause OA, such as inflammatory arthritis (e.g. rheumatoid and psoriatic arthritis), bone diseases (e.g. Paget), skeletal dysplasias, nutritional deficiencies (Kashin-Beck and rickets) and metabolic disturbances (e.g. ochronosis and hemochromatosis). In these less common cases, OA can occur prematurely and lead to the failure of the joint if early diagnosis is not made and/or specific treatment is not currently available, such as for skeletal dysplasias. In other similarly atypical cases, often with a strong family history, however, no clear causes can be identified using readily available tests.

Genetic and genome-wide association studies (GWAS) have shown the importance of genetic factors in the risk for the development of OA, suggesting that 40–65% of this risk may be due to genetic susceptibility after adjustment for common OA risk factors such as age, sex and body mass index [5, 6]. Several common variants have been identified by GWAS, although the contribution of each individual variant to the risk for OA development is only moderate-to-small, with consistent associations found for the growth and differentiation factor-5 (GDF-5) gene [7]. Familial cases of OA can provide an opportunity to further understand and characterize less common OA etiologies so that more targeted treatment strategies can be developed as well as providing a greater understanding of mechanisms towards disease onset.

The aim of this study was to investigate a family with multiple members affected by early-onset OA, including its clinical, biochemical and imaging manifestations and genetics associations, and to develop an evaluation strategy including a clinical pathway and bioinformatics pipeline that, if established, could be applied to other families affected by early-onset OA. A clinical description of the condition affecting this family has been published previously [8]; however, no laboratory investigations were performed at that time.

## Methods

### Setting and participants

This study was conducted at the Royal North Shore Hospital, the Children’s Hospital at Westmead and the Garvan Institute of Medical Research, New South Wales, Australia. Participants were recruited from a family with multiple members affected by OA before 40 years of age (herein referred as early-onset OA) and involving one or more joints. Eligible participants for this study were members of the index family with or without a history of OA. Participants who were not able to travel to the study centre received the study documentation and questionnaires via email or post.

### Procedures

An invitation letter was sent to members of the index family. We tried to be as inclusive as possible and contacted individuals from different generations. Those who expressed interest in participating in the study were contacted by the study coordinator either by phone or email and received the participant information sheet. Potential participants were invited to attend a face-to-face visit at the study center and had the opportunity to discuss any questions related to the study with the study coordinator. All participants (and/or legal guardian) were asked to provide their written consent before data collection. For participants undergoing genetic testing, a genetic counsellor was involved in consenting the participants for the study.

Participants were asked to complete electronic questionnaires containing demographic and clinical information. This included history of OA symptoms, age of onset, past medical history and use of medications, joints affected, symptom severity and associated symptoms and history of prior joint surgery. We obtained written consent from participants to access previous imaging of affected joints.

**Fig. 1:**
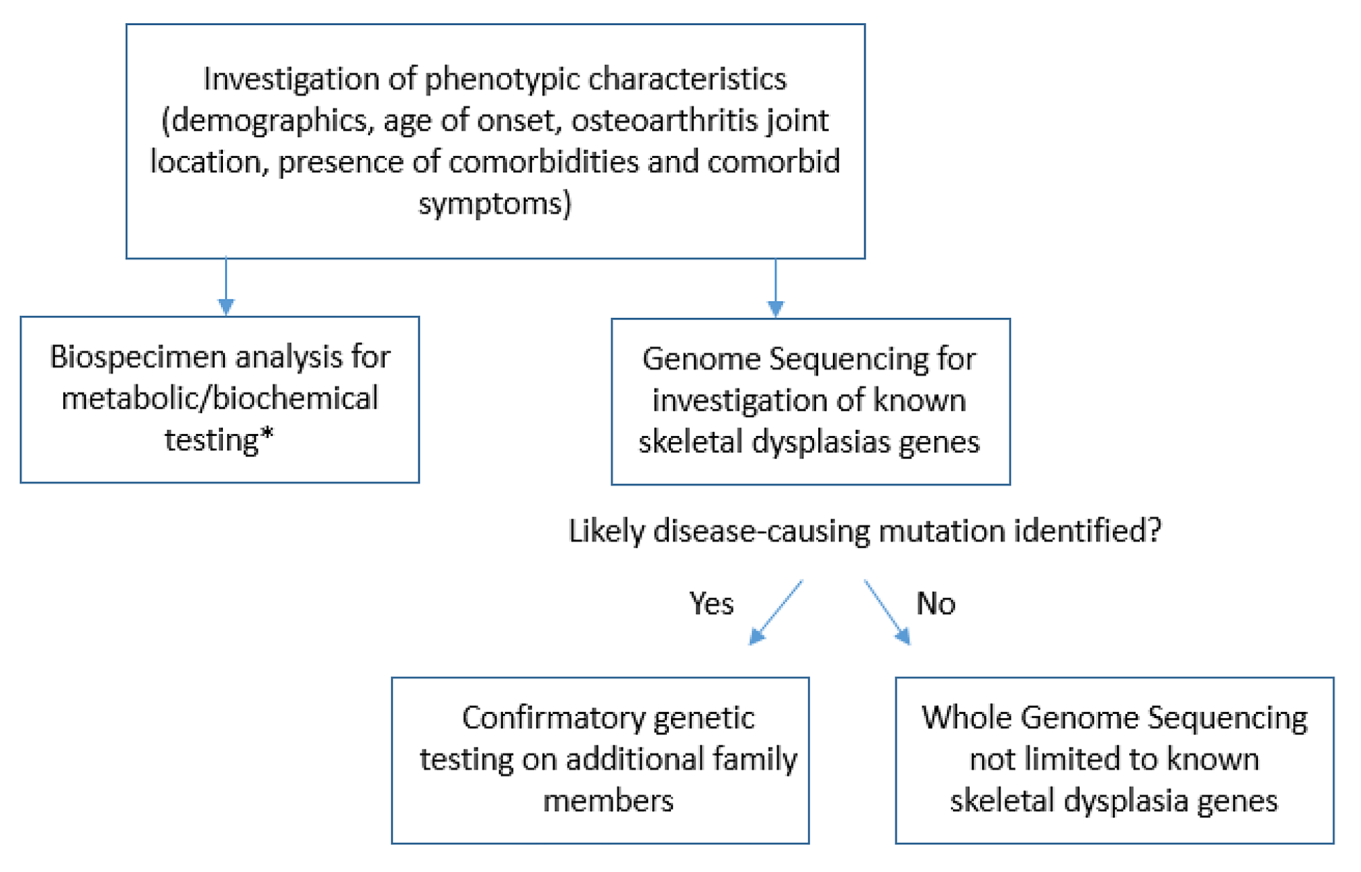
Workflow for the investigation of the early-onset OA in the family *see Table 1

A workflow for the investigation of the early onset OA in the family is displayed in Fig. 1.

### Imaging analysis

We obtained copies of participants’ relevant joint imaging for characterization of the extent of OA and the presence of unusual features such as the presence of calcium deposits and shape abnormalities. The images were reviewed by two rheumatologists (LD and DH) and a musculoskeletal radiologist. Images were also reviewed by a clinical geneticist (AZ) for features of skeletal dysplasia.

### Biochemical and genetic testing

Blood and urine samples were obtained from a subsample of participants (n = 10) for biochemical analysis to investigate diseases associated with OA (Table 1). Participants referred for biochemical testing were selected mostly based on their availability. Biochemical testing was performed in participants affected and not affected by the condition to serve as controls. Case status (affected vs. unaffected) was self-reported by the participants during the initial screening.

**Table 1:** Laboratory investigation of potential diseases associated with the development of early-onset osteoarthritis

Condition	Laboratory test
Paget	Serum alkaline phosphatase
Acromegaly	Serum IGF-1
Kashin-Beck	Selenium (blood)
Vitamin D deficiency	Serum 25-hydroxyvitamin D [25-(OH)D]
Inflammatory arthritis	CRP, ESR
Gout	Serum uric acid
Thyroidopathy	TSH
Diabetes Mellitus	HbA1C
Hemochromatosis	Serum transferrin saturation
Hyperparathyroidism	PTH
Wilson disease	Serum ceruloplasmin, 24-hour urinary excretion of copper
Hemoglobinopathy	Hemoglobin electrophoresis
Hypophosphatasia	Serum phosphorus
Hypomagnesemia	Serum magnesium

### Genetic testing

**Fig. 2:**
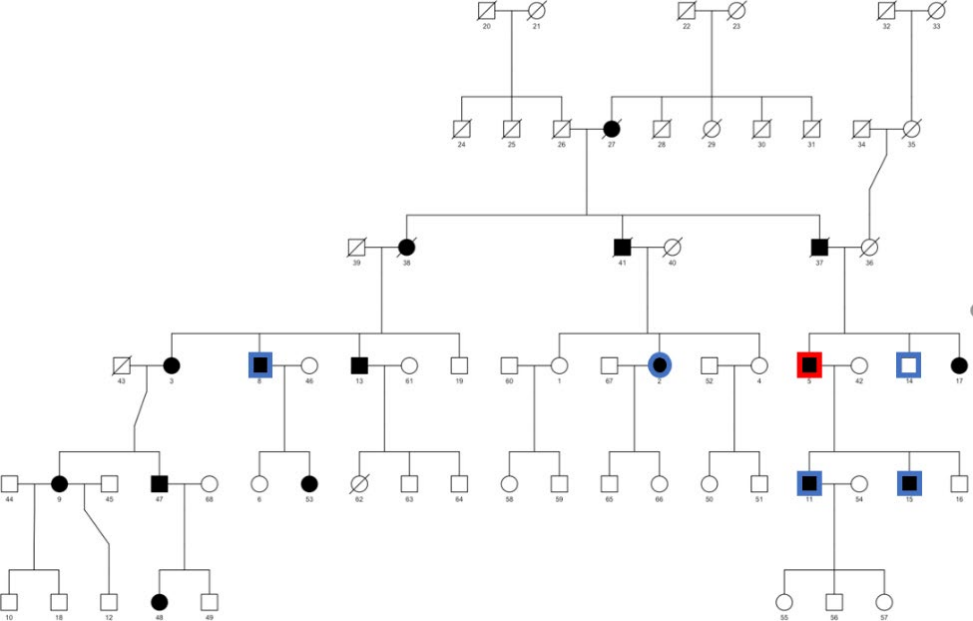
Pedigree chart displaying the family tree and showing members of the family affected by early-onset OA. Filled symbols mean affected, unfilled symbols mean unaffected. Crossed symbols mean deceased. Whole Genome Sequencing was performed in one family member, indicated in red, and confirmatory tests were performed in another 5 family members (4 cases and 1 control), indicated in blue

Whole Genome Sequencing was performed on DNA extracted from peripheral blood in one family member affected by early-onset OA, selected based on clinical characteristics and participant availability (see Fig. 2 below). Whole genome sequencing was performed using a modified KAPA Hyper PCR-free Library Preparation kit and Illumina 150 bp paired-end sequencing. Paired-end reads are aligned to the human genome reference sequence (GRCh37) using the Burrows-Wheeler Aligner (BWA-MEM), and variant calls are made using the Genomic Analysis Tool Kit (GATK). Analysis was restricted to the genes listed in the 2019 Revision of the Nosology and Classification of Genetic Skeletal Disorders [9]. Single nucleotide and small indel variants were classified according to the joint consensus recommendations of the American College of Medical Genetics and Genomics and the Association for Molecular Pathology [10]. Confirmatory genetic testing on the index case and additional family members (four affected by early-onset OA and one non-affected) was performed by Sanger Sequencing.

## Results

Table 2 describes the clinical characteristics of the 17 participants included in the study. There was an even split between the two sexes and two participants were under 18 years old. Eleven participants (65%) reported the presence of joint symptoms, while four (23%) had no symptoms in their joints and two (12%) had previously experienced joint symptoms. The shoulders, hands and ankles were the most commonly affected joints. Amongst all of the participants, 18 joints have been replaced. The most common areas were the hips (n = 8) and knees (n = 6).

**Table 2:** Clinical and demographic characteristics of the participants (n = 17)

	Cases (n = 9)	Controls (n = 8)
**Demographics**		
Age, mean (range)	60 (40–74)	43 (10–75)
Age < 18 years, n (%)	0 (0)	2 (100)
Female sex, n (%)	4 (44)	4 (50)
BMI, mean (SD)	27 (3)	23 (3)
Height (cm), mean (SD)	175 (8)	170 (12)
Weight (kg), mean (SD)	84 (13)	69 (13)
Smoking status, n (%)		
Never	4 (44)	6 (75)
In the past	5 (55)	2 (25)
Current	0 (0)	0 (0)
**Clinical characteristics**		
Presence of joint symptoms, (%)		
Yes	9 (100)	2 (25)
No	0 (0)	4 (50)
In the past	0 (0)	2 (25)
Joints affected, n (%)		
Knee(s)	6 (66)	1 (12)
Hip(s)	2 (22)	1 (12)
Shoulder(s)	9 (100)	0 (0)
Neck	5 (55)	0 (0)
Lower back	4 (44)	0 (0)
Wrist(s)	5 (55)	0 (0)
Finger(s)	8 (88)	1 (12)
Elbow(s)	6 (66)	0 (0)
Ankle(s)	8 (88)	0 (0)
Foot/Feet	5 (55)	0 (0)
Jaw(s)	0 (0)	0 (0)
Joints replaced or fused, n (%)		
Knee	5 (55)	1 (12)
Hip	8 (88)	0 (0)
Shoulder	1 (11)	0 (0)
Lumbar spine	1 (11)	0 (0)
Ankle	2 (22)	0 (0)
Foot/Neck/Wrist(s)/Finger(s)/Elbow(s)	0 (0)	0 (0)
Duration of symptoms, n (%)		
Less than 1 year	0 (0)	2 (25)
1–5 years	0 (0)	0 (0)
5–10 years	0 (0)	0 (0)
> 10 years	9 (100)	1 (12)
Osteoarthritis diagnosis, n (%)		
Yes	9 (100)	1 (12)
No	0 (0)	2 (25)
Pain during past 24 hours*		
(0-100), mean (SD)	44 (18)	1 (2)
Joint pain frequency, n (%)		
Monthly	0 (0)	2 (25)
Weekly	1 (11)	1 (12)
Daily	5 (55)	0 (0)
Always	3 (33)	0 (0)
Use of pain medications, n (%)		
Paracetamol	8 (88)	1 (12)
NSAIDs	9 (100)	2 (25)
Opioids	6 (66)	0 (0)
Use of supplements, n (%)		
No	6 (66)	6 (75)
In the past	3 (33)	0 (0)
Currently	0 (0)	2 (25)

*Numerical rating scale

Ten participants (77%) reported longstanding symptoms (over ten years) and have already received the diagnosis of OA. The mean pain average (numerical rating scale, NRS) was 31 out of 100 (100 being the worst pain possible) and the use of pain medication was common.

A pedigree diagram was constructed to illustrate occurrence of the condition in multiple generations of the family (Fig. 2). The pedigree structure suggested that the early-onset OA in the family could be inherited in an autosomal dominant fashion.

### Imaging

Radiographs from six affected participants were reviewed, including images of the spine (n = 5), hip (n = 4), ankle (n = 3), knee (n = 2), elbow (n = 2), shoulder (n = 2) and feet (n = 1). There were common features of OA in the joints, including osteophyte formation, joint space loss and subchondral sclerosis. Less typical features that were present were metaphyseal enlargement, pistol grip deformity in the hips, joint surface irregularities and involvement of the second and third metatarsophalangeal joints.

Screening for potential diseases associated with early-onset OA.

Laboratory screening for potential causes of the early-onset OA in the family was performed in 7 cases and 3 controls (Table 3). Cases more often had elevated alkaline phosphatase, C-reactive protein, uric acid and PTH compared to controls. There was one isolated case of elevated IGF-1 in a participant affected by early-onset OA. However, no pattern of abnormalities that could explain the OA phenotype in the family was observed.

**Table 3:** Laboratory investigation

	Cases (n = 7), n (%) within normal range*	Controls (n = 3), n (%) within normal range
Serum alkaline phosphatase	4 (57)	3 (100)
Serum IGF-1	6 (85)	3 (100)
Selenium (blood)	7 (100)	3 (100)
Serum 25-hydroxyvitamin D [25-(OH)D]	7 (100)	3 (100)
CRP	5 (71)	3 (100)
Serum uric acid	4 (57)	3 (100)
TSH, free T4	7 (100)	3 (100)
HbA1C	7 (100)	3 (100)
Serum transferrin saturation and ferritin concentration	7 (100)	3 (100)
PTH	5 (71)	3 (100)
Serum ceruloplasmin	7 (100)	3 (100)
24-hour urinary excretion of copper**		
Hemoglobin electrophoresis	7 (100)	3 (100)
Serum phosphorus	7 (100)	3 (100)
Serum magnesium	7 (100)	3 (100)

*All abnormal results were above the normal values

**24-hour urinary excretion of copper was low in 3 cases and 1 control and normal in 1 case. Other participants did not collect 24h urine

### Genetic analysis

Whole Genome Sequencing identified a heterozygous variant in the *COL2A1* gene in one affected family member:

COL2A1 (NM_001844.4): c.[2155 C > T];[2155=] p.[(Arg719Cys)];[Arg719=]

This variant (sometimes referred to as p.Arg519Cys in older literature) is very rare in the general population but has been identified in several other families with early-onset osteoarthritis [11–13]. The variant was classified as pathogenic according to established guidelines [10]. The finding was confirmed by Sanger Sequencing in an accredited molecular genetics diagnostic laboratory (Children’s Hospital at Westmead, NSW, Australia) in a separate sample of the same participant. Subsequently, DNA from five additional participants (four affected and one unaffected) was analysed for this mutation. The same mutation was identified in the four participants affected by OA, while it was not present in the unaffected individual. Taken together, this variant was thought to be associated with the development of early-onset OA in the family.

## Discussion

Large scale genome-wide association studies (GWAS) have found several risk variants associated with OA. At present, more than one hundred DNA polymorphisms have been described, highlighting the polygenic nature of OA [14]. Polymorphisms have been identified in a range of different alleles, most commonly modifying the regulation of gene expression as opposed to changing protein structure [15]. Post-GWAS studies focused on understanding the biological (or pathological) function of risk variants have been increasingly used to translate genetic discovery into useful research information, particularly for the development of new therapeutic targets. In fact, proteins encoded by OA-associated genes identified by GWAS are the target of new potential disease-modifying OA drugs, including Wnt inhibitors and anabolic agents such as fibroblast-growth factor 18. In contrast, in a small percentage of the identified polymorphisms, the alteration is in a gene coding sequence with amino acid substitution that leads to abnormal protein function. This is the case of familial mutations in the type II collagen gene (*COL2A1*) [16], as identified in this family.

Type II collagen is a major constituent of articular cartilage. This variant in the type II collagen gene leads to the substitution of an arginine with a cysteine molecule in the triple helix domain of type II collagen. Functional studies suggest that this leads to abnormal assembly of type II collagen fibrils [17]. These abnormal collagen fibrils are probably less resistant to wear and tear and thus lead to early onset of OA, though the exact disease mechanisms are still unknown. Different arginine to cysteine mutations in the *COL2A1* gene have been described as causing a spectrum of clinical phenotypes [18], including spondyloepiphyseal dysplasia congenita, spondyloarthropathy, abnormalities in digits and Stickler dysplasia [11]. In our study, affected individuals were slightly taller and heavier than controls and had OA involving multiple joints including spine, large joints and fingers. There were no cases of hearing, vision or orofacial abnormalities or shorter digits. Available radiographs showed some features consistent with type 2 collagenopathy, such as metaphyseal enlargement and epiphyseal irregularities in addition to common OA changes.

The prevalence and disease burden caused by early-onset OA has been increasing progressively since 1990, largely driven by rising obesity rates in younger adults [19, 20]. In a population-based study from the United States, the prevalence of early-onset OA has been estimated to be 0.74% and 0.88% among non-obese men and women, respectively, between 25 and 34 years and 1.74% and 2.06% among those between 35 and 44 years [2]. History of joint trauma/injury or significant joint malalignment are other important causes of early-onset OA. Cases of early-onset OA in the absence of a clear predisposing risk factor, such as skeletal dysplasias, are rare although the exact prevalence is still not well established [21]. Ruault et al. performed next-generation sequencing in 45 individuals with early-onset OA and identified a genetic variant in 29% of patients, most commonly in the *COL2A1* gene [22]. Despite the enormous burden caused by these conditions in those affected, particularly due to the early age of onset and typical chronic course of pain and disability, they remain incredibly restricted in the reported research. In these cases, investigation of underlying OA-associated disorders is important, particularly when other members of the family are also affected. There is a broad range of disorders that have been associated with early-onset OA and, often, no other obvious clinical manifestation is present. Early recognition of underlying disorders is vital since specific treatments are available for many conditions, such as in the case of inflammatory arthritis and haemochromatosis, and for genetic counselling, in case of skeletal dysplasias. Currently, there is no clear guidance on how best to investigate families affected by early-onset OA, thus we aimed to provide an evaluation strategy to identify potential OA-associated disorders.

In this study, we included laboratory tests to investigate a wide selection of potential causes of early-onset OA, as shown in Table 1. However, this list can be tailored depending on the phenotypic characteristics of the family members obtained in the first step of the clinical assessment. For example, the Kashin-Beck disease has a specific geographic distribution, primarily affecting individuals in China, Siberia and North Korea. Patients without a positive epidemiology would not need to be screened for this disease. We propose that sequencing of known skeletal dysplasia genes should be performed as the initial step for investigation of pathogenic variants, with whole genome sequencing reserved for cases where no diagnosis has been established after initial laboratory and genetic screening. If a likely underlying OA-associated disease is found, confirmatory tests in other family members (affected and non-affected) should be performed to confidently correlate the identified disease to the OA phenotype in the family.

The main limitation of this study is the inclusion of only one family to test the performance of the proposed evaluation strategy for the investigation of familial cases of early-onset OA. Further studies would be useful to assess the performance and cost-effectiveness of this strategy. Also, the affected/unaffected status was self-reported and, as we did not perform the laboratory and genetic investigation in all participants due to logistic reasons, it is possible that not all participants affected by OA have the identified mutation in the *COL2A1* gene. Lastly, all cases reported joint symptoms for more than 10 years and it is possible, although unlikely, that laboratory markers would have been different in earlier OA stages.

In conclusion, the methodology used in this study, including the clinical pathway and bioinformatics pipeline, could be applied to other families affected by early-onset OA. Future studies should focus on estimating the prevalence of skeletal dysplasias and other OA-associated diseases in early-onset OA and investigating the patient and disease characteristics predictive of presence of pathogenic variants in this population to help with early recognition and diagnosis of these diseases.

## Data Availability

The datasets generated and/or analysed during the current study are available in the ClinVar repository, accession number SCV003842221.1.
